# Investigations of Possible Multistate Outbreaks of *Salmonella*, Shiga Toxin–Producing *Escherichia coli*, and *Listeria monocytogenes* Infections — United States, 2016

**DOI:** 10.15585/mmwr.ss6906a1

**Published:** 2020-11-13

**Authors:** Katherine E. Marshall, Thai-An Nguyen, Michael Ablan, Megin C. Nichols, Misha P. Robyn, Preethi Sundararaman, Laura Whitlock, Matthew E. Wise, Michael A. Jhung

**Affiliations:** ^1^Division of Foodborne, Waterborne, and Environmental Diseases, National Center for Emerging and Zoonotic Infectious Diseases, CDC; ^2^Oak Ridge Institute for Science and Education

## Abstract

**Problem/Condition:**

*Salmonella*, Shiga toxin–producing *Escherichia coli* (STEC), and *Listeria monocytogenes* are the leading causes of multistate foodborne disease outbreaks in the United States. Responding to multistate outbreaks quickly and effectively and applying lessons learned about outbreak sources, modes of transmission, and risk factors for infection can prevent additional outbreak-associated illnesses and save lives. This report summarizes the investigations of multistate outbreaks and possible outbreaks of *Salmonella*, STEC, and *L. monocytogenes* infections coordinated by CDC during the 2016 reporting period.

**Period Covered:**

2016. An investigation was considered to have occurred in 2016 if it began during 2016 and ended on or before March 31, 2017, or if it began before January 1, 2016, and ended during March 31, 2016–March 31, 2017.

**Description of System:**

CDC maintains a database of investigations of possible multistate foodborne and animal-contact outbreaks caused by *Salmonella*, STEC, and *L.* *monocytogenes.* Data were collected by local, state, and federal investigators during the detection, investigation and response, and control phases of the outbreak investigations. Additional data sources used for this report included PulseNet, the national molecular subtyping network based on isolates uploaded by local, state, and federal laboratories, and the Foodborne Disease Outbreak Surveillance System (FDOSS), which collects information from state, local, and territorial health departments and federal agencies about single-state and multistate foodborne disease outbreaks in the United States. Multistate outbreaks reported to FDOSS were linked using a unique outbreak identifier to obtain food category information when a confirmed or suspected food source was identified. Food categories were determined and assigned in FDOSS according to a classification scheme developed by CDC, the Food and Drug Administration (FDA), and the U.S. Department of Agriculture Food Safety and Inspection Service (FSIS) in the Interagency Food Safety Analytics Collaboration.

A possible multistate outbreak was determined by expert judgment to be an outbreak if supporting data (e.g., temporal, geographic, demographic, dietary, travel, or food history) suggested a common source. A solved outbreak was an outbreak for which a specific kind of food or animal was implicated (i.e., confirmed or suspected) as the source. Outbreak-level variables included number of illnesses, hospitalizations, cases of hemolytic uremic syndrome (HUS), and deaths; the number of states with illnesses; date of isolation for the earliest and last cases; demographic data describing patients associated with a possible outbreak (e.g., age, sex, and state of residence); the types of data collected (i.e., epidemiologic, traceback, or laboratory); the outbreak source, mode of transmission, and exposure location; the name or brand of the source; whether the source was suspected or confirmed; whether a food was imported into the United States; the types of regulatory agencies involved; whether regulatory action was taken (and what type of action); whether an outbreak was publicly announced by CDC via website posting; beginning and end date of the investigation; and general comments about the investigation. The number of illnesses, hospitalizations, cases of HUS, and deaths were characterized by transmission mode, pathogen, outcome (i.e., unsolved, solved with suspected source, or solved with confirmed source), source, and food or animal category.

**Results:**

During the 2016 reporting period, 230 possible multistate outbreaks were detected and 174 were investigated. A median of 24 possible outbreaks was under investigation per week, and investigations were open for a median of 37 days. Of these 174 possible outbreaks investigated, 56 were excluded from this analysis because they occurred in a single state, were linked to international travel, or were pseudo-outbreaks (e.g., a group of similar isolates resulting from laboratory media contamination rather than infection in patients). Of the remaining 118 possible multistate outbreaks, 50 were determined to be outbreaks and 39 were solved (18 with a confirmed food source, 10 with a suspected food source, 10 with a confirmed animal source, and one with a suspected animal source). Sprouts were the most commonly implicated food category in solved multistate foodborne outbreaks (five). Chicken was the source of the most foodborne outbreak-related illnesses (134). Three outbreaks involved novel food–pathogen pairs: flour and STEC, frozen vegetables and *L. monocytogenes*, and bagged salad and *L. monocytogenes*. Eleven outbreaks were attributed to contact with animals (10 attributed to contact with backyard poultry and one to small turtles). Thirteen of 18 multistate foodborne disease outbreaks with confirmed sources resulted in product action, including 10 outbreaks with recalls, two with market withdrawals, and one with an FSIS public health alert. Twenty outbreaks, including 11 foodborne and nine animal-contact outbreaks, were announced to the public by CDC via its website, Facebook, and Twitter. These announcements resulted in approximately 910,000 webpage views, 55,000 likes, 66,000 shares, and 5,800 retweets.

**Interpretation:**

During the 2016 reporting period, investigations of possible multistate outbreaks occurred frequently, were resource intensive, and required a median of 37 days of investigation. Fewer than half (42%) of the 118 possible outbreaks investigated were determined to have sufficient data to meet the definition of a multistate outbreak. Moreover, of the 50 outbreaks with sufficient data, approximately three fourths were solved.

**Public Health Action:**

Close collaboration among CDC, FDA, FSIS and state and local health and agriculture partners is central to successful outbreak investigations. Identification of novel outbreak sources and trends in sources provides insights into gaps in food safety and safe handling of animals, which helps focus prevention strategies. Summarizing investigations of possible multistate outbreaks can provide insights into the investigative process, improve future investigations, and help prevent illnesses. Although identifying and investigating possible multistate outbreaks require substantial resources and investment in public health infrastructure, they are important in determining outbreak sources and implementing prevention and control measures.

## Introduction

Each year in the United States, *Salmonella*, Shiga toxin–producing *Escherichia coli* (STEC), and *Listeria monocytogenes* infections cause an estimated 1.49 million illnesses, 28,000 hospitalizations, and 700 deaths (*1*) and cause approximately $6 billion in costs of illness (*2*). These three pathogens are the leading causes of multistate foodborne disease outbreaks in the United States (*3*,*4*). Multistate outbreaks of these infections typically are associated with contaminated food products or contact with live animals or pets that are widely distributed and sold. Although multistate outbreaks comprise 3% of all foodborne outbreaks, they account for one third of hospitalizations and approximately half of foodborne illness-related deaths (*4*). Multistate outbreaks comprise approximately half of outbreaks associated with animal contact, which are of particular concern because they disproportionately affect young children, a population at increased risk for severe illness (*5*). Responding to multistate outbreaks quickly and effectively can prevent additional outbreak-associated illnesses and save lives. Lessons learned during outbreak investigations about sources, modes of transmission, and risk factors for infection provide insights to help prevent illnesses and deaths.

CDC leads a national network of local, state, and federal public health officials who investigate possible multistate outbreaks of *Salmonella*, STEC, *L. monocytogenes*, and other enteric infections. For each possible outbreak, CDC facilitates rapid and coordinated responses by working with state and local health officials and federal agencies to determine whether enough data exist to suggest that the group of illnesses constitutes an outbreak, investigate to identify the source of the outbreak, recommend actions to stop the outbreak, and communicate findings to the public and food, backyard poultry, and companion animal industries when actions to prevent additional illnesses can be taken.

This report summarizes the investigations of possible multistate outbreaks of *Salmonella*, STEC, and *L. monocytogenes* infections coordinated by CDC and conducted by local and state health departments, laboratories, and environmental and agriculture agencies during the 2016 reporting period. Calculations of the proportions of possible outbreaks that were classified as outbreaks and those that were solved and summaries of the solved outbreaks are included. Identified gaps in food safety and lessons learned can focus efforts that might help prevent future outbreaks. This report provides the public and food, backyard poultry, and companion animal industries with information about CDC’s multistate outbreak investigation process, the multistate outbreaks that were investigated, and the foods and animals that were common sources. This information can be used by local, state, and federal public health practitioners, academic partners, and partners in the food, backyard poultry, and companion animal industries to help develop outbreak and illness preventive interventions.

## Methods

### Multistate Investigation Process

CDC coordinates multistate investigations that have four main phases. These phases are 1) detection (i.e., identification of a possible multistate outbreak), 2) assessment (i.e., determining whether CDC will coordinate and help investigate), 3) investigation and response (i.e., collecting data to determine the outbreak source), and 4) control (i.e., taking action to stop the outbreak, including communicating about the outbreak publicly, and assisting federal regulatory agencies including the Food and Drug Administration [FDA] and the U.S. Department of Agriculture Food Safety and Inspection Service [FSIS] in product recalls and market withdrawals).

#### Detection Phase

Possible multistate outbreaks (also referred to as clusters) were identified from three primary sources: 1) PulseNet, the national molecular subtyping network based on isolates uploaded by local, state, and federal laboratories (*6*), 2) local or state health departments, and 3) FDA and FSIS. The PulseNet database team identified possible outbreaks by looking for increases in a particular pulsed-field gel electrophoresis pattern over time (*7*,*8*). The investigation process began when one of these sources identified a possible outbreak and notified epidemiologists at CDC.

#### Assessment Phase

CDC epidemiologists reviewed and assessed initial information available in PulseNet about possible multistate outbreaks of *Salmonella* and STEC infections to determine whether to investigate. Typically, all possible outbreaks of *L.* *monocytogenes* infections that were detected were investigated because of their severity. Primary factors considered in these assessments included size (total number of illnesses) and scope (geographic distribution of illnesses); how frequently and recently new illnesses were being reported and whether they were being reported more frequently than expected on the basis of a cumulative sum algorithm (*9*); how common the strain was; whether the same strain was recently isolated from food, animal, or environmental sources or previously linked to an outbreak; demographic characteristics of patients; and which resources were available to dedicate to an investigation. Reasons for not investigating possible multistate outbreaks included unlikelihood of gathering enough quality exposure data to identify a source (e.g., a long period from onset of illness to recognition of possible outbreak), illnesses caused by a strain that was not reported more frequently than expected because they likely represented sporadic illness, illnesses that appeared to be associated with international travel, or exposures that occurred in a single state. Possible outbreaks with illnesses occurring in a single state were investigated and coordinated by state or local health departments rather than CDC.

#### Investigation and Response Phase

After an investigation began, CDC, local and state health and agriculture departments, FDA’s Coordinated Outbreak Response and Evaluation Network (https://www.fda.gov/food/outbreaks-foodborne-illness/about-core-network), and FSIS’s Office of Public Health Science, Applied Epidemiology Staff (https://www.fsis.usda.gov/wps/portal/fsis/home) collected epidemiologic, traceback, and laboratory data to try to identify and confirm an outbreak source. Epidemiologic data were collected from interviews with patients using state-specific questionnaires or standard national questionnaires (https://www.cdc.gov/foodsafety/outbreaks/surveillance-reporting/investigation-toolkit.html) that include many questions about typical exposures (e.g., the National Hypothesis Generating Questionnaire and the *Listeria* Initiative questionnaire). Epidemiologic data were collected using the System for Enteric Disease Response, Investigation, and Coordination (SEDRIC), a web-based system developed by CDC and Palantir Technologies (Denver, Colorado) to streamline and coordinate outbreak investigations (https://www.cdc.gov/foodsafety/outbreaks/investigating-outbreaks/sedric.html). Investigators assessed epidemiologic data to determine whether illnesses were likely to be caused by a common source and what that source was likely to be. Typically, when a suspected source was identified and purchase information was available from patients, a traceback investigation was conducted to try to identify a common source in the distribution chain. If a common source was identified, investigators gathered additional information by conducting inspections on farms or in facilities that manufacture, process, pack, or hold food. When warranted, investigators also attempted to collect samples from food, animals, or the environment of facilities where food and animals are grown or raised, processed, or sold.

#### Control Phase

When data collected implicated a specific food product, companies could conduct a market withdrawal or a product recall. In certain cases, regulatory agencies enforced mandatory recalls of product from the implicated company. In other cases, the company chose to voluntarily initiate a market withdrawal or product recall. When an outbreak source was identified and consumers or industry could take actions to prevent additional illnesses, local and state health departments, CDC, and federal regulatory agencies alerted the public and industry through website postings, social media including Twitter and Facebook, and press releases.

### Definitions and Variables

A possible multistate outbreak was defined as a group of ill persons, living in two or more states, who were infected with the same bacterial strain of *Salmonella*, STEC, or *L. monocytogenes* reported to CDC. An outbreak strain was defined as a group of isolates with indistinguishable pulsed-field gel electrophoresis patterns, isolates that were highly related to one another by whole genome sequencing, or both. An outbreak-related case was defined as infection with an outbreak strain in a person identified during the investigation period. The specific time frame for possible outbreaks identified by PulseNet included illnesses that initially were reported within the past 60 days (*Salmonella* and STEC infections) or the past 120 days (*L. monocytogenes* infections) of the date the possible outbreak was detected. A possible multistate outbreak was determined by expert judgment to be an outbreak if supporting data (e.g., temporal, geographic, demographic, dietary, travel, and food history) suggested a common source. A solved outbreak was an outbreak for which a specific kind of food or animal was implicated (i.e., confirmed or suspected) as the source.

Outbreak sources were classified as suspected or confirmed on the basis of the strength of supporting epidemiologic, traceback, and laboratory data collected during the investigation. Typically, a source was classified as suspected if it was implicated only by epidemiologic data (i.e., the data supporting it as the source of an outbreak were only epidemiologic in nature). A source was considered confirmed if it was implicated by epidemiologic plus traceback or laboratory data (https://www.cdc.gov/foodsafety/outbreaks/investigating-outbreaks/index.html). Epidemiologic data included 1) a higher than expected proportion of patients with exposure to the same food or animal before illness onset and 2) two or more unrelated patients who ate at the same restaurant, shopped at the same grocery store, or attended the same event. To determine whether a higher than expected proportion of patients was exposed to a particular food or animal, investigators frequently used techniques such as a binomial model to compare the proportion of patients associated with an outbreak with healthy persons who participated in the FoodNet Population Survey (*10*,*11*). Traceback data included shipping, purchase, or other information that suggested a common point of contamination in the distribution chain of food products or animals (*12*). Laboratory data included isolation of the outbreak strain from a suspected source (food or animal) or the environment around the source (environment in which the animal lived or area where food was grown, processed, or sold). Transmission mode (i.e., foodborne or animal contact) was determined only for outbreaks and not possible outbreaks.

Possible actions resulting from outbreaks included recalls (a company’s removal of product from commerce to protect the public from adverse health consequences), market withdrawals (a company’s removal of product from commerce when the product has minor quality issues or a minor regulatory violation), and public health alerts (public notification by a regulatory agency about a product that might be associated with human illnesses when a recall could not be recommended or a company was unwilling to perform a recall).

### Data Sources

The primary data source used to generate this report was a CDC database maintained by the Outbreak Response and Prevention Branch (ORPB) containing outbreak-level data, including aggregated patient-level data, gathered during the investigation (Table 1). These data were collected by local, state, and federal investigators during each phase of the investigation and entered into the database. During the detection phase, information was entered into the database principally from PulseNet and included the source of the report, the date a possible outbreak was identified, the total number of outbreak-related illnesses at that time, states with illnesses, pathogens involved (including strain designation), date of isolation for the earliest and most recent cases, demographic data describing patients associated with a possible outbreak (e.g., age, sex, and state of residence), and a unique identifier for each possible outbreak, assigned by PulseNet. Assignment of outbreak status (i.e., an outbreak or a cluster) also was recorded in the database. During the investigation and response phase, the following outbreak-level data were documented: the number of hospitalizations and deaths; the types of data collected (i.e., epidemiologic, laboratory, or traceback); the outbreak source, mode of transmission, and exposure location; the name or brand of the source; whether the source was suspected or confirmed; whether a food was imported into the United States; the types of regulatory agencies involved; whether regulatory action was taken (and what type of action); whether an outbreak was publicly announced by CDC via website posting; end date of the investigation; and general comments about the investigation.

**TABLE 1:** Primary and supplemental data sources for investigations of possible multistate enteric disease outbreaks — United States, 2016

Data source	Description of data source	Data type	Unit of analysis	Variables collected
Outbreak Response and Prevention Branch (ORPB) database	ORPB maintains a database that contains data collected by local, state, and federal investigators during each phase of an active outbreak investigation	Primary	Outbreak level (including aggregated patient data)	Source of the report; date possible outbreak identified; total number of illnesses; states with illnesses; unique identifier for the outbreak; pathogen; strain; the date of isolation for the earliest and most recent case; percent female and age range for ill persons; total number of persons who were hospitalized, diagnosed with hemolytic uremic syndrome, or died; type of data collected; outbreak source; mode of transmission; location of exposure to the source; name or brand of the source; whether the source was the suspected or confirmed source; whether the source was imported into the United States; types of regulatory agencies involved; whether regulatory action was taken and what type of action; whether the outbreak was announced by CDC; end date of the investigation; and general comments
PulseNet	PulseNet is a molecular subtyping network for foodborne bacterial disease surveillance	Supplemental	Case level	Patient’s age, sex, and state of residence
Foodborne Disease Outbreak Surveillance System (FDOSS)	FDOSS is a surveillance system for single-state and multistate foodborne outbreaks in the United States	Supplemental	Outbreak level	Food category

Supplemental data sources included the PulseNet database (*6*) and the Foodborne Disease Outbreak Surveillance System (FDOSS), which collects information from state, local, and territorial health departments, and federal agencies about single-state and multistate foodborne outbreaks in the United States. Multistate outbreaks captured in the ORPB database were reported to FDOSS and linked using a unique outbreak identifier to obtain food category information when a confirmed or suspected food source was identified. Food categories were determined and assigned in FDOSS based on a classification scheme developed by CDC, FDA, and FSIS in the Interagency Food Safety Analytics Collaboration (*13*).

### Inclusion Criteria

All possible multistate outbreaks of *Salmonella*, STEC, and *L. monocytogenes* infections identified and reported to ORPB during the 2016 reporting period were included in this analysis. An investigation was considered to have occurred in 2016 if it began during 2016 and ended on or before March 31, 2017, or if it began before January 1, 2016, and ended during March 31, 2016–March 31, 2017.

### Exclusion Criteria

Certain possible multistate outbreak investigations that CDC coordinated and assisted with were excluded from this report. These included outbreaks that initially were thought to be multistate outbreaks but later were identified as primarily single-state outbreaks during the investigation phase, those that were identified as associated with international travel, and those that were determined to be pseudo-outbreaks (e.g., a group of similar isolates resulting from laboratory media contamination rather than infection in patients).

### Analysis

Outbreak duration was calculated as the number of days between the first and last dates the outbreak strain was isolated from patients. The investigation duration was calculated as the number of days between CDC’s identification of a possible outbreak and when the CDC coordination and investigation ended. All outbreaks, illnesses, hospitalizations, cases of hemolytic uremic syndrome (HUS), and deaths were analyzed by transmission mode, pathogen, outcome (i.e., unsolved, solved with suspected source, or solved with confirmed source), source, and food or animal category.

## Results

During the 2016 reporting period, CDC assessed 230 possible multistate outbreaks of infections caused by *Salmonella*, STEC, and *L. monocytogenes*; 200 were detected by PulseNet, 25 by state and local health departments, and five by FDA and FSIS. Of these 230 possible outbreaks, 174 were investigated, 50 were determined to be outbreaks, and 39 were solved. Of the 56 possible multistate outbreaks that were not investigated, 25 appeared to be single-state outbreaks, 19 had illnesses caused by a strain that was not reported more frequently than expected, six had a majority of illnesses that occurred too far in the past to gather enough quality exposure data to identify a source, three appeared to be associated with international travel, and three did not have a reason recorded.

**FIGURE 1:**
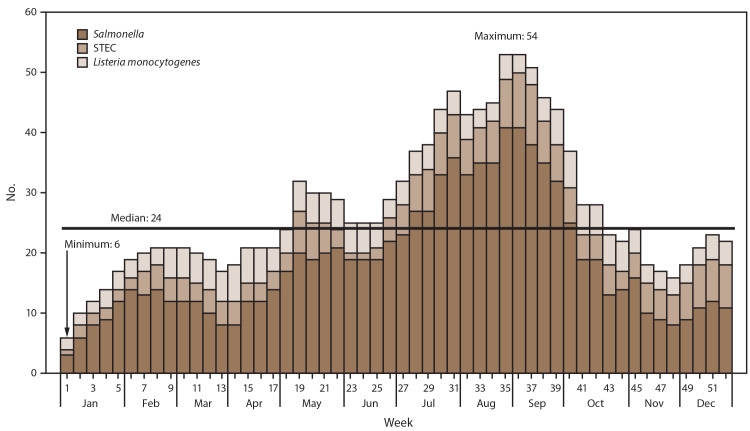
Number of ongoing possible multistate outbreak investigations,* by pathogen and week — United States, 2016 **Abbreviation:** STEC = Shiga toxin–producing *Escherichia coli.* * n = 174.

CDC facilitated the investigation of 174 possible outbreaks of *Salmonella* (120), STEC (38), and *L. monocytogenes* (16) infections. A median of 24 possible outbreaks was under investigation per week (Figure 1), and the highest number of investigations per week (53) occurred during the last week of August and first week of September 2016. The lowest number of investigations per week (six) occurred in the first week of January 2016. The median duration for investigations of these 174 possible outbreaks was 37 days. Of these 174 possible outbreaks investigated, 56 were excluded from this analysis: 41 were associated with exposure in a single state, 13 were associated with international travel, and two were pseudo-outbreaks (data suggesting an outbreak but no clinical illnesses actually occurred [e.g., when contaminated culture media causes clinical specimens to falsely appear to contain bacteria]).

The remaining 118 possible multistate outbreaks were associated with 3,480 illnesses, 752 hospitalizations, 16 cases of HUS, and 26 deaths (Table 2). Investigators did not identify information suggesting a common source for 68 (58%) of these 118 possible multistate outbreaks; the remaining 50 (42%) were classified as outbreaks. Among these 50 outbreaks, 39 (78%) were solved, including 11 outbreaks with a suspected source and 28 outbreaks with a confirmed source. Investigations of solved outbreaks lasted longer than investigations of unsolved outbreaks (median: 86 versus 70 days). A higher proportion of *Salmonella* outbreaks was solved (30 of 34), compared with *L. monocytogenes* (four of six) and STEC outbreaks (five of 10). As a result of these investigations, 10 product recalls, two market withdrawals, and one FSIS public health alert were issued. CDC communicated to the public about 20 of these outbreaks to warn consumers to take action to reduce risks for illness (e.g., avoid eating implicated foods; https://www.cdc.gov/outbreaks.index.html).

**TABLE 2:** Characteristics of multistate outbreaks of *Listeria monocytogenes,* Shiga toxin–producing *Escherichia coli*, and *Salmonella* infections, by implicated source (food, animal contact, or unknown) and pathogen — United States, 2016

Variable	Solved multistate outbreaks (a common food source was implicated)				Solved multistate outbreaks (contact with a common animal was implicated as the source)		Unsolved multistate outbreaks (common source unknown)				All multistate outbreaks (N = 50)
	*L. monocytogenes* (n = 4)	STEC (n = 5)	*Salmonella* (n = 19)	Total (n = 28)	*Salmonella* (n = 11)	Total (n = 11)	*L. monocytogenes* (n = 2)	STEC (n = 5)	*Salmonella* (n = 4)	Total (n = 11)	
**Outbreak characteristic**											
**Duration, days**											
Median (range) duration of outbreak	495 (152–963)	65 (24–259)	73 (17–218)	81 (17–963)	160 (36–607)	160 (36–607)	202 (58–345)	22 (4–83)	72 (18–136)	59 (4–345)	**86 (4–963)**
Median (range) duration of investigation	131 (49–207)	39 (23–245)	69 (25–195)	70 (23–245)	141 (39–512)	141 (39–512)	267 (95–438)	49 (18–77)	74 (69–79)	70 (18–438)	**78 (18–512)**
**Size**											
No. of cases	39	102	515	656	1,068	1,068	43	70	87	200	**1,929**
Median (range) per outbreak	9 (2–19)	11 (11–56)	28 (6–70)	14 (2–70)	86 (14–248)	86 (14–248)	22 (5–38)	13 (4–26)	19 (5–45)	16 (4–45)	**20 (2–248)**
**Geographic distribution**											
No. of states with cases	17	30	46	50	49	49	12	19	23	35	**50**
Median (range) per outbreak	6 (2–9)	5 (2–24)	9 (2–24)	8 (2–24)	25 (8–40)	25 (8–40)	7 (3–10)	5 (2–14)	7 (2–19)	6 (2–19)	**9 (2–39)**
**Disposition**											
Outbreak: confirmed source	3 (75)	3 (60)	12 (63)	18 (64)	10 (91)	10 (91)	—*	—	—	—	**28 (72)**
Outbreak: suspected source	1 (25)	2 (40)	7 (37)	10 (36)	1 (9)	1 (9)	—	—	—	—	**11 (28)**
**Patient characteristic**											
**Age**											
Median age (range), yrs	70 (<1–91)	20 (1–95)	32 (<1–94)	31 (<1–95)	25 (<1–106)	25 (<1–106)	73 (29–93)	24 (2–98)	43 (<1–93)	42 (<1–98)	**30 (<1–106)**
No. (%) of ill children aged <5 yrs	3/38 (8)	10/101 (10)	53/510 (10)	66/649 (10)	389/1,050 (37)	389/1,050 (37)	3/40 (8)	6/68 (9)	7/86 (8)	16/194 (8)	**388/1,898 (20)**
No. (%) of patients aged >65 yrs	23/38 (61)	9/101 (9)	62/510 (12)	94/649 (14)	128/1,050 (12)	128/1,050 (12)	26/40 (65)	11/68 (16)	17/86 (20)	54/194 (28)	**276/1,898 (15)**
**Sex**											
Median (range) % female per outbreak	62 (38–80)	55 (45–77)	64 (36–82)	63 (36–82)	53 (42–79)	53 (42–79)	34 (20–47)	65 (50–92)	66 (51–88)	60 (20–92)	**59 (20–92)**
**Outcome**											
No. of hospitalizations	36	37	98	171	248	248	36	21	16	73	**492**
Median (range) % hospitalized per outbreak	100 (75–100)	36 (18–70)	25 (0–43)	29 (0–100)	28 (11–40)	28 (11–40)	91 (82–100)	38 (0–77)	29 (19–50)	40 (0–100)	**29 (0–100)**
No. (range) of deaths per outbreak	7 (1–3)	0	1	8 (0–3)	3 (0–2)	3 (0–2)	3 (1–2)	3 (0–2)	0	6 (0–2)	**17 (0–3)**
**Strain characteristic**											
Serogroup/serotype/pathogen (no. of outbreaks caused)^†^	1/2a (3) 1/2b (1) 4b (1)	O157 (4) O121 (1) O26 (1)	*S.* Enteritidis (4) *S.* Saintpaul (2) *S.* Typhimurium (2) *S.* Abony (1) *S.* Anatum (1) *S.* Braenderup (1) *S.* Goldcoast (1) I,4,[5],12:i:– (1) *S.* Javiana (1) *S.* Minnesota (1) *S.* Montevideo (1) *S.* Muenchen (1) *S.* Granienburg (1) *S.* Oslo (1) *S.* Reading (1) *S.* Senftenberg (1) *S.* Virchow (1)		*S.* Braenderup (2) *S.* Infantis (2) *S.* Enteritidis (1) *S.* Hadar (1) *S.* Indiana (1) *S.* Mbandaka (1) *S.* Muenster (1) *S.* Ohio (1) *S.* Poona (1) *S.* Pomona (1) I,4,[5],12,b– (1) Iiib 61:i:z53 (1)		1/2b (1) 4b (1)	O157 (3) O5 (1)	*S.* Javiana (2) *S.* Bareilly (1) *S.* Sundsvall (1)		

**Abbreviations:** *L. monocytogenes* = *Listeria monocytogenes*; *S*. = *Salmonella*; STEC = Shiga toxin–producing *Escherichia coli*.

* Not applicable.

^†^ >1 serotype/serogroup could be included in an outbreak.

### Multistate Outbreaks Linked to Food Sources

Among the 39 solved multistate outbreaks, 28 (72%) were linked to contaminated foods during the 2016 reporting period. These 28 outbreaks were associated with 656 illnesses, 171 hospitalizations, five cases of HUS, and eight deaths (Table 2). A total of 19 foodborne outbreaks were caused by *Salmonella*, five by STEC, and four by *L. monocytogenes*. Outbreaks of *Salmonella* were larger in both geographical scope (involving a total of 46 states [median: 9; range: 2–24]) and size (515 total patients) than outbreaks of STEC (30 states; median: 5; range: 2–24; 102 total patients) and *L. monocytogenes* (17 states; median: 6; range: 2–9; 39 total patients). However, *L. monocytogenes* infections were more severe, resulting in a higher median proportion of hospitalizations (100%) per outbreak than either STEC (36%) or *Salmonella* (25%) infections, and more deaths (seven) than *Salmonella* (one) or STEC (zero) infections. The median age of patients linked to a foodborne disease outbreak was 31 years. Patients in outbreaks of STEC infections were younger than those in outbreaks of *Salmonella* infections (median: 20 years versus 32 years). The median age for persons in outbreaks of *L. monocytogenes* infections was 70 years, reflecting the population primarily affected by invasive listeriosis (https://www.cdc.gov/listeria). Although the median duration of all investigations was 37 days, the median duration of investigations of solved foodborne outbreaks was 70 days. The duration of solved *L. monocytogenes* outbreaks (median: 495 days) and the duration of their investigations (median: 131 days) were also much longer than the duration of solved *Salmonella* outbreaks (median: 73 days) and investigations (median: 69 days) or solved STEC outbreaks (median: 65 days) and investigations (median: 39 days).

Sprouts were the most commonly implicated food in multistate foodborne outbreaks (five) during the 2016 reporting period and were associated with the second-most outbreak-related illnesses (131) (Table 3). Although contaminated chicken was the source of fewer multistate outbreaks (two) than sprouts, it resulted in the most outbreak-related illnesses (134). The source of the largest multistate foodborne outbreak investigated was chicken, which resulted in 70 illnesses and seven hospitalizations. In 2016, ready-to-eat foods (i.e., foods that do not require consumers to cook them before eating them) were the source of more outbreaks and outbreak-related illnesses than foods not considered ready to eat (20 versus eight outbreaks; 412 versus 244 illnesses). Four outbreaks were linked to foods that were imported from other countries (hot peppers, cucumbers, and melons imported from Mexico and Persian-variety cucumbers imported from an undetermined country).

**TABLE 3:** Multistate outbreaks and related illnesses caused by *Salmonella* species*,* Shiga toxin–producing *Escherichia coli*, and *Listeria monocytogenes*, by food or animal source — United States, 2016

Source	*Salmonella*		STEC		*L. monocytogenes*		Total	
	Outbreaks	Illnesses	Outbreaks	Illnesses	Outbreaks	Illnesses	Outbreaks No. (%)	Illnesses No. (%)
Beef	—*	—	1	11	—	—	**1 (4)**	**11 (2)**
Ground beef^†^	—	—	1	11	—	—	**1**	**11**
Chicken	2	134	—	—	—	—	**2 (7)**	**134 (20)**
Chicken	2	134	—	—	—	—	**2**	**134**
Dairy	—	—	—	—	1	2	**1 (4)**	**2 (<1%)**
Raw milk	—	—	—	—	1	2	**1**	**2**
Eggs	1	8	—	—	—	—	**1 (4)**	**8 (1)**
Eggs^†^	1	8	—	—	—	—	**1**	**8**
Fruits	2	69	—	—	—	—	**2 (7)**	**69 (11)**
Avocado	1	59	—	—	—	—	**1**	**59**
Melon^§^	1	10	—	—	—	—	**1**	**10**
Grains/Beans	—	—	2	69	—	—	**2 (7)**	**69 (11)**
Flour^†^	—	—	1	56	—	—	**1**	**56**
Pizza dough	—	—	1	13	—	—	**1**	**13**
Herbs	1	35	—	—	—	—	**1 (4)**	**35 (5)**
Powdered supplement^†^	1	35	—	—	—	—	**1**	**35**
Multiple	1	28	—	—	3	37	**4 (14)**	**65 (10)**
Salad mix	1	28	—	—	—	—	**1**	**28**
Bagged salad^†^	—	—	—	—	1	19	**1**	**19**
Frozen vegetables^†^	—	—	—	—	1	10	**1**	**10**
Hummus	—	—	—	—	1	8	**1**	**8**
Nuts/Seeds	2	17	—	—	—	—	**2 (7)**	**17 (3)**
Hazelnuts	1	6	—	—	—	—	**1**	**6**
Pistachios^†^	1	11	—	—	—	—	**1**	**11**
Pork	1	12	—	—	—	—	**1 (4)**	**12 (2)**
Pork	1	12	—	—	—	—	**1**	**12**
Root/Underground vegetables	1	29	—	—	—	—	**1 (4)**	**29 (4)**
Onions	1	29	—	—	—	—	**1**	**29**
Seeded vegetables	3	56	—	—	—	—	**3 (11)**	**56 (9)**
Cucumbers^†,§^	1	10	—	—	—	—	**1**	**10**
Hot peppers^§^	1	32	—	—	—	—	**1**	**32**
Persian cucumbers^§^	1	14	—	—	—	—	**1**	**14**
Sprouts	4	120	1	11	—	—	**5 (18)**	**131 (20)**
Alfalfa sprouts^†,§^	1	36	1	11	—	—	**2**	**47**
Bean sprouts	2	52	—	—	—	—	**2**	**52**
Other sprouts	1	32	—	—	—	—	**1**	**32**
Vegetable row crops	1	7	1	11	—	—	**2 (7)**	**18 (3)**
Iceberg lettuce	—	—	1	11	—	—	**1**	**11**
Leafy greens	1	7	—	—	—	—	**1**	**7**
**Total foods**	**19**	**516**	**5**	**106**	**4**	**39**	**28 (72)**	**656 (38)**
Backyard poultry	10	930	—	—	—	—	**10**	**930**
Turtles	1	138	—	—	—	—	**1**	**138**
**Total animals**	**11**	**1,068**	**—**	**—**	**—**	**—**	**11 (28)**	**1,068 (62)**
**Total outbreaks and illnesses**	**30**	**1,584**	**5**	**106**	**4**	**39**	**39 (100)**	**1,724 (100)**

**Abbreviations:** *L. monocytogenes* = *Listeria monocytogenes*; STEC = Shiga toxin–producing *Escherichia coli*.

* No multistate outbreak investigations coordinated by CDC were linked to this source.

^†^ Recalled.

^§^ Imported.

**FIGURE 2:**
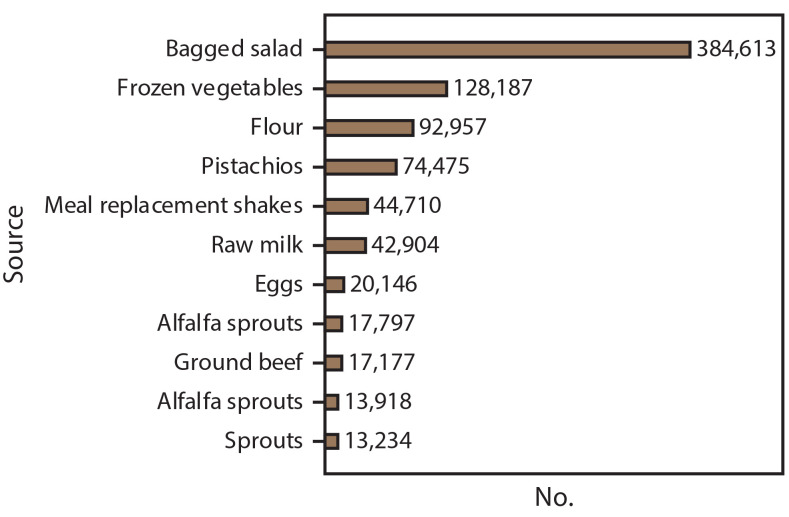
Number of webpage views for CDC announcements of multistate foodborne outbreaks,* by outbreak source — United States, 2016 * n = 11.

Of 18 multistate foodborne outbreaks with confirmed sources, 13 resulted in product action, including 10 outbreaks with recalls, two with voluntary market withdrawals but no recalls, and one with an FSIS public health alert. CDC announced 11 foodborne outbreaks to the public via its website, Facebook, and Twitter, resulting in approximately 850,000 webpage views, 50,000 likes, 62,000 shares, and 5,000 retweets. The three outbreak website postings that had the most page views included two *L. monocytogenes* outbreaks linked to bagged salad (384,613 views) and frozen vegetables (128,187), followed by an STEC outbreak linked to flour (92,957) (Figure 2). The outbreaks that had the least page views were the two *Salmonella* outbreaks linked to sprouts and alfalfa sprouts (13,234 and 13,918, respectively) and STEC outbreaks linked to ground beef and alfalfa sprouts (17,177 and 17,797, respectively).

### Multistate *Salmonella* Outbreaks Linked to Food Sources

Among the 30 solved *Salmonella* outbreaks, 19 (63%) were linked to contaminated food. The most common serotype was *Salmonella* Enteritidis (four outbreaks), followed by *Salmonella* serotype Saintpaul and *Salmonella* serotype Typhimurium (two each) (Table 2). These 19 outbreaks were associated with 515 illnesses, 98 hospitalizations, and one death across 46 states. Sprouts (four outbreaks, 120 illnesses), and chicken (two outbreaks, 134 illnesses) were the most commonly identified *Salmonella* outbreak sources. Of these 19 *Salmonella* outbreaks, eight led to product actions, including five recalls, two market withdrawals, and one FSIS public health alert.

### Multistate STEC Outbreaks Linked to Food Sources

All five solved STEC outbreaks were linked to contaminated food. STEC O157 caused the most outbreaks (four); one outbreak was caused by both STEC O121 and O26 (Table 2). These five outbreaks resulted in 106 illnesses, 37 hospitalizations, and five cases of HUS across 30 states. In 2016, multistate STEC foodborne outbreaks were linked to grains or beans (two outbreaks, 73 illnesses), beef (one outbreak, 11 illnesses), sprouts (one outbreak, 11 illnesses), and vegetable row crops (one outbreak, 11 illnesses). The sources of the multistate STEC outbreaks categorized as grains or beans were contaminated flour and pizza dough containing contaminated flour (Table 3). Of the five outbreaks, three led to product recalls.

### Multistate *L. monocytogenes* Outbreaks Linked to Food Sources

Each of the four solved multistate listeriosis outbreaks was linked to contaminated food. These four outbreaks were responsible for 39 illnesses, 36 hospitalizations, and seven deaths across 17 states (Table 2). *L.* *monocytogenes* outbreaks were linked to multiple sources, including bagged salad, frozen vegetables, hummus, and dairy (raw milk). This was the first multistate outbreak of *L. monocytogenes* infections linked to raw milk. Two outbreaks resulted in product recalls.

### Multistate Outbreaks Linked to Contact with Animals

Among the 39 solved multistate outbreaks, 11 (28%) were linked to contact with animals; all were caused by *Salmonella* (Table 2). Ten outbreaks were attributed to contact with backyard poultry and one outbreak to contact with small turtles (carapace <4 inches) (Table 3). These 11 outbreaks resulted in 1,068 illnesses, 248 hospitalizations, and three deaths across 49 states. The median age of patients from all animal-contact outbreaks was 25 years. Children aged <5 years (37%) made up a higher proportion of patients in multistate outbreaks linked to contact with animals compared with foodborne outbreaks (10%). The duration of animal-contact outbreaks (median: 160 days) and their investigations (median: 141 days) was longer than the duration of foodborne outbreaks (median: 81 days) and their investigations (median: 70 days). Nine outbreaks linked to contact with animals were announced to the public via website, Facebook, and Twitter, resulting in approximately 61,000 page views, 4,000 likes, 4,000 shares, and 600 retweets.

### Unsolved Multistate Outbreaks

Eleven of the 50 multistate outbreaks were unsolved and included four outbreaks of *Salmonella* infections, five outbreaks of STEC infections, and two outbreaks of *L. monocytogenes* infections (Table 2). These 11 outbreaks resulted in 200 illnesses, 73 hospitalizations, and six deaths. Data collected to determine the source of 10 unsolved outbreaks were suggestive of a common food source; however, data were not sufficient to implicate a specific food item or animal source.

## Discussion

Investigations of possible multistate outbreaks occurred frequently during the 2016 reporting period, with a median of 24 active investigations ongoing each week. These outbreak investigations were resource intensive, requiring a median of 37 days of investigation. During the 2016 reporting period, 230 possible outbreaks were detected, 174 were investigated, 50 were determined to be outbreaks, and 39 were solved, which led to immediate actions to prevent additional illness, including 10 food product recalls, two market withdrawals, one FSIS public health alert, and CDC website postings for 20 foodborne and animal-contact outbreaks. The investigative work also informed long-term prevention strategies and provided useful information to industry partners and consumers.

Investigating multistate outbreaks of enteric illness can be complex and challenging at each stage of the investigation. In 2016, approximately half of the 118 possible outbreaks were determined by investigators to have insufficient data to meet the definition of a multistate outbreak. This often occurred because investigators were either unable to collect enough information about cases to suggest a credible common exposure existed or whole genome sequencing did not substantiate a common exposure that pulsed-field gel electrophoresis suggested existed. Identifying the source of outbreaks also poses challenges; 22% of outbreaks investigated in 2016 did not have a source identified. In general, persons often have difficulty remembering foods that they ate before they became ill because they often are interviewed long after their exposure occurred. Limited resources at local and health state departments could result in patients not being interviewed, and, even if all patients are interviewed, missing or limited interview data from patients can hamper source identification. For example, in 2016, among 10 Foodborne Disease Centers for Outbreak Response and Enhancement (FoodCORE) centers in local and state health departments that received resources to improve completeness and timeliness of foodborne disease outbreak responses (*14*), exposure information was unavailable for 8.8%–14.2% of patients with *Salmonella*, STEC, and *L. monocytogenes* infections. Among patients for whom exposure information was available, 6.6%–24.7% had only a short, initial interview completed, meaning the information was limited in scope and detail, although this has improved over time (https://www.cdc.gov/foodcore/metrics/ssl-metrics.html).

Patients with listeriosis are often older and too ill to be interviewed; therefore, obtaining complete questionnaires for them is especially challenging. Even when longer interviews are completed, investigators still might not inquire about obscure, unusual, or infrequent exposures. Although 400 food and 42 animal exposures are included in the National Hypotheses Generating Questionnaire and approximately 100 food exposures are included in the *Listeria* Initiative questionnaire, this still places a cap on the number of possible exposure sources that can be explored by standard interviews. Investigators can conduct open-ended interviews to ask questions about exposure to other foods and animals, although these are time-consuming for both interviewers and patients. However, open-ended interviews and other approaches (e.g., examination of shopper card records) have been used with success to identify new outbreak sources and solve outbreaks (*11*,*15*,*16*). Finally, a suspected outbreak source was identified in 28% of multistate investigations in 2016; however, sources were not confirmed because of insufficient traceback or laboratory data. Challenges in the traceback and laboratory investigations include a lack of traceable consumer information (e.g., receipts or shopper card history) identified from interviews with patients, a lack of food or animals available for testing, and a lack of detailed records along the food distribution chain for traceback. Detailed records are required for traceback, including receipts, bills of lading, and other sale documents.

Among solved foodborne outbreaks of *Salmonella*, STEC, and *L. monocytogenes* infections in 2016, sprouts were the most common source (five outbreaks). Sprouts were the source of both STEC and *Salmonella* outbreaks and were associated with the second-highest number of outbreak-related cases (131), following chicken (134). CDC’s announcements of three separate outbreaks linked to sprouts in 2016 garnered less attention from consumers compared with outbreaks associated with other food products. The relatively low number of page views might reflect the smaller proportion of persons who eat sprouts compared with other foods (*10*); however, additional research would help to better understand consumer knowledge of the risks associated with consuming raw sprouts and whether outbreak messages about sprouts reach persons who eat them. In addition, the FDA Food Safety Modernization Act (FSMA) Produce Safety Rule includes new requirements for sprouts producers to help prevent the contamination of sprouts (*17*).

In 2016, multistate outbreaks of *Salmonella* infections linked to contact with animals caused more illnesses than multistate outbreaks of all pathogens linked to contaminated food (1,068 versus 656 illnesses). In addition, more illnesses were linked to contact with backyard poultry in 2016 (930 illnesses) than in any previous year (*18*,*19*). This might be explained partly by the increasing popularity of keeping backyard poultry in the United States (*20*). The 10 outbreaks linked to contact with backyard poultry caused eight times as many illnesses as the two outbreaks linked to consumption of chicken in 2016. This finding is unexpected because a survey of healthy persons conducted in 2006 indicated that 65% of persons in the United States ate chicken, whereas <3% of persons were exposed to live chickens in the previous week (*10*). Reasons for more *Salmonella* infections linked to backyard poultry than consumption of chicken are unclear; however, this might be related to differences in ascertainment of outbreaks attributed to each transmission mode and the risk for infection posed by each.

Foodborne outbreaks linked to chicken often are challenging to investigate because chicken meat is eaten so frequently (*10*), and patients often do not remember specific information (e.g., brands, purchase location, and lot codes) needed to confirm chicken as an outbreak source. In contrast, outbreaks linked to contact with backyard poultry might be more straightforward to investigate because exposure to live poultry is less common, although increasing (*10*,*20*), and relatively limited information (e.g., whether a patient keeps backyard poultry) is needed to identify the source. Although a greater number of cases was associated with backyard poultry compared with consumed chicken among solved outbreaks, the number of cases attributed to each transmission mode among unsolved outbreaks is unknown. Risk for *Salmonella* infection might result from both direct and indirect exposure to backyard poultry (and areas where the birds live and roam, which can be contaminated with *Salmonella*); therefore, more opportunities for pathogen transmission might exist. Taken together, the number of outbreaks and outbreak-related illnesses linked to contact with backyard poultry and consumed chicken indicates that carriage of *Salmonella* by poultry and its transmission to persons is considerable.

In 2016, three outbreaks were noteworthy because they involved novel food–pathogen pairs. For the first time, STEC was definitively linked to raw flour, although investigators have suspected flour in several previous outbreaks (*21*), including an earlier 2016 STEC outbreak linked to pizza dough (*22*). Lessons learned from this outbreak include confirmation that STEC can survive in low-moisture foods such as flour, which typically do not support bacterial growth, and can cause illness in persons exposed to contaminated flour. This outbreak also demonstrated that both consumption of raw or undercooked dough and playing with raw dough are risk factors for illness. In response to this outbreak, and to address gaps in consumer knowledge about risks associated with flour and dough, CDC advised consumers to avoid eating or tasting any unbaked products that are intended to be baked and to not allow children to play with or eat raw dough (*15*,*23*). Ruminants, especially cattle, are the primary reservoir for STEC. Contamination with STEC might start in the wheat fields and lead to contamination of harvested wheat. Flour producers are researching ways to reduce contamination in flour to prevent future outbreaks (*24*,*25*).

Two *L. monocytogenes* outbreaks were linked to novel outbreak sources for this pathogen: one to frozen vegetables (*26*) and one to bagged salad (*27*). Both foods were widely consumed, and the resulting product recalls were large (frozen produce: 456 products sold under 42 brands and 47 million pounds of meat and chicken meals containing frozen produce; bagged salad: 22 products sold under six U.S. and two Canadian brands) and costly (bagged salad: $25.5 million) (*27*–*29*). At the time of the outbreak, frozen produce was not considered to be ready to eat, meaning that consumers were expected to cook their frozen produce to kill any pathogens that might be present, including *L. monocytogenes*. Information about how ill persons prepared their frozen vegetables was available for one person included in the outbreak and a group of ill persons not included in the outbreak. In this group of persons who ate recalled frozen vegetables and had gastrointestinal symptoms compatible with noninvasive infection with *L. monocytogenes*, a clinical specimen from one person did not yield *L. monocytogenes*. Of the two persons for whom information was available about how they prepared frozen vegetables, one reported cooking frozen vegetables on the stove and the other reported eating them both raw and cooked. This suggests persons might be thawing and eating frozen produce without cooking it to a temperature that destroys pathogens. To address these gaps in the food safety system, more research on consumer knowledge, attitudes, and practices regarding the risks associated with eating uncooked or undercooked frozen produce might be useful. In addition, FDA is developing prevention strategies for ready-to-eat foods, recognizing that not all frozen vegetables are considered traditionally ready to eat. In addition, industry and academic partners have been conducting research on preventing *L. monocytogenes* contamination of leafy greens (*30*) and in facilities that manufacture frozen produce and bagged salad.

## Limitations

The findings in this report are subject to at least five limitations. First, the primary data source, the ORPB database, was not designed to be a surveillance system with ongoing, systematic collection and analysis of outbreak-level data. Possible multistate outbreaks might not have been captured in the ORPB database because they were not detected or because they were identified by a federal, state, or local agency; assessed informally by CDC without an investigation; and not subsequently entered into the database. Most single-state outbreaks are not captured by the ORPB database because these outbreaks typically are investigated and coordinated by state or local health departments and might be reported to FDOSS but not ORPB. Therefore, the number of single-state outbreaks reported in this report is a substantial underrepresentation of all that occur. Second, FDOSS does not use the same methods used to generate this report; therefore, the number of multistate outbreaks reported to FDOSS might be different than the number reported in this report. Third, these findings are not a complete description of the number, distribution, or etiology of all outbreak-associated *Salmonella*, STEC, and *L. monocytogenes* illnesses in the United States. For many multistate outbreaks, inclusion of a patient in the outbreak depended on culture confirmation of their infection, and case totals do not include the far greater number of ill persons who might have been part of the outbreak but did not seek care or have specimens cultured. Moreover, the number of outbreaks and illnesses attributed to specific foods might not reflect those of all single-state or multistate outbreaks that occurred during the 2016 reporting period. Fourth, the decision to classify a possible outbreak as an outbreak was made on the basis of expert opinion, and misclassification might have occurred. Finally, the number of patients linked to recognized outbreaks likely represents a small proportion of all laboratory-confirmed *Salmonella*, STEC, and *L. monocytogenes* illnesses, and the sources of illnesses not associated with outbreaks might be different from those associated with outbreaks.

## Future Directions

The findings in this report establish a baseline to help assess changes in outbreak detection, investigation, and response after implementation of new technologies for subtyping and other improvements in traceback and outbreak communications. The introduction of whole genome sequencing as the standard method for molecular subtyping in PulseNet in mid-2019 is likely to provide more information about outbreaks than previously known. By using whole genome sequencing, rather than pulsed-field gel electrophoresis, to detect and define possible outbreaks, investigators are likely to detect possible outbreaks while they are smaller, which might increase the likelihood that investigations will be more productive (*31*). Enhanced detection, investigation, and prevention efforts should result in fewer illnesses and outbreaks and greater confidence in the effectiveness of prevention strategies. Meeting these challenges in the future will take dedicated effort and support at the local, state, and national levels.

Each outbreak investigation is an opportunity to better understand the reasons why the outbreak occurred, identify immediate control measures, and identify longer-term prevention measures. Combined with other surveillance and analytic studies, the lessons learned in outbreak investigations can help focus improved prevention efforts. Preventing more foodborne disease will depend on the combined efforts of many partners and stakeholders. Since 2018, CDC has enhanced efforts to work with partners to identify more targets for interventions, fill gaps in the scientific knowledge base, and promote, implement, and evaluate strategies for the prevention of enteric disease outbreaks. Preventing enteric disease illnesses and outbreaks, including those caused by *Salmonella*, STEC, and *L. monocytogenes*, requires research, educational campaigns, and collaboration with many partners in public health, regulatory agencies, academia, industry, and consumer groups.

## Conclusion

This report describes CDC’s multistate investigation process; summarizes possible multistate outbreaks of *Salmonella*, STEC, and *L. monocytogenes* infections that were detected, assessed, and investigated during the 2016 reporting period; highlights investigation challenges and lessons learned; and identifies gaps in the food safety system. Close collaboration among federal agencies and state and local health and agriculture partners is central to successful outbreak investigations, and multistate investigations could not be conducted without the work performed by local and state health, laboratory, and regulatory agencies. Identifying and investigating possible multistate outbreaks require substantial federal, state, and local resources to identify outbreak sources, implement control steps, and develop improved prevention measures.

Identification of novel outbreak sources and trends in sources provides insights into gaps in food safety and safe handling of animals that help focus prevention strategies. Sprouts were identified as the source of most multistate foodborne disease outbreaks, and backyard poultry was identified as the most common source of multistate animal-contact outbreaks. Several novel food–pathogen combinations caused outbreaks in 2016, including STEC in flour and *L. monocytogenes* in bagged salad and frozen produce.

Summarizing investigations of possible multistate outbreaks can provide insights into the investigative process, improve future investigations, and help prevent illnesses. Finally, this report also highlights the importance of comprehensive, evidence-based practices, processes, and regulations to detect and investigate multistate outbreaks of enteric illness. Such practices result in better information about outbreaks, including what causes them and which solutions end them quickly. This information, in turn, helps CDC and partners in the food, backyard poultry, and companion animal industries, government, and public health develop strategies to help mitigate the impact of these outbreaks and ideally prevent them in the future.
